# Characterizing the Propagation of Uterine Electrophysiological Signals Recorded with a Multi-Sensor Abdominal Array in Term Pregnancies

**DOI:** 10.1371/journal.pone.0140894

**Published:** 2015-10-27

**Authors:** Diana Escalona-Vargas, Rathinaswamy B. Govindan, Adrian Furdea, Pam Murphy, Curtis L. Lowery, Hari Eswaran

**Affiliations:** 1 Department of Obstetrics and Gynecology, University of Arkansas for Medical Sciences, Little Rock, Arkansas, United States of America; 2 Division of Fetal and Transitional Medicine, Children’s National Medical Center, Washington, DC, United States of America; 3 Institute for Medical Psychology and Behavioral Neurobiology, University of Tuebingen, Tuebingen, Germany; PreTel Inc, UNITED STATES

## Abstract

The objective of this study was to quantify the number of segments that have contractile activity and determine the propagation speed from uterine electrophysiological signals recorded over the abdomen. The uterine magnetomyographic (MMG) signals were recorded with a 151 channel SARA (SQUID Array for Reproductive Assessment) system from 36 pregnant women between 37 and 40 weeks of gestational age. The MMG signals were scored and segments were classified based on presence of uterine contractile burst activity. The sensor space was then split into four quadrants and in each quadrant signal strength at each sample was calculated using center-of-gravity (COG). To this end, the cross-correlation analysis of the COG was performed to calculate the delay between pairwise combinations of quadrants. The relationship in propagation across the quadrants was quantified and propagation speeds were calculated from the delays. MMG recordings were successfully processed from 25 subjects and the average values of propagation speeds ranged from 1.3–9.5 cm/s, which was within the physiological range. The propagation was observed between both vertical and horizontal quadrants confirming multidirectional propagation. After the multiple pairwise test (99% CI), significant differences in speeds can be observed between certain vertical or horizontal combinations and the crossed pair combinations. The number of segments containing contractile activity in any given quadrant pair with a detectable delay was significantly higher in the lower abdominal pairwise combination as compared to all others. The quadrant-based approach using MMG signals provided us with high spatial-temporal information of the uterine contractile activity and will help us in the future to optimize abdominal electromyographic (EMG) recordings that are practical in a clinical setting.

## Introduction

Over past years there have been several attempts to better understand the electrophysiological activity of uterine contractile mechanism both in animals and humans. The uterine contractions are a manifestation of complex electrophysiological process and have a direct impact on the labor process and subsequent delivery of the fetus. The need to investigate the electrophysiological mechanism of the uterus arises from the fact that there is a lack of truly effective method of diagnosis and management of labor. Better insight into the mechanism can provide more objective assessment of the labor process that can result in effective and timely therapeutic interventions to alleviate issues surrounding both normal and preterm labor.

The uterine electromyogram (EMG) is one of the techniques that has been applied for over fifty years using both internal electrodes and abdominal surface electrodes to track the changes of the uterus going from a quiescent phase to high state of activation, increased propagation and synchronization of electrical activity across the whole organ [1–15]. In early studies Steer et al [1–2] and Sureau et al [13] have tried to map the topography of the electrical activity of the uterus using multiple electrodes. Steer et al [1–2] placed two pairs of electrodes overlying each fallopian tube junction and placed a third pair high in the mid-line of the fundus. They reported that a weak activity picked by one of the two pairs of electrodes showed a small time lag in early labor and the lag diminished as the labor progressed. During labor they observed that the activity from all the three pairs of electrodes were almost simultaneous. These studies show that the progress of labor is related to the propagation of electrical activity throughout the uterus.

More recently, the importance of electrical propagation has been shown in work by Lammers et al [15] where they measured electrical potential from the serosa of isolated pregnant uteri using large number of silver electrodes (> 200) and recording from these simultaneously. From published data in rats [16–17] and the work on the whole uterus in guinea pigs [15], it has been shown that action potentials propagate in a specific manner along the uterine wall, with rapid conduction in the longitudinal and slow conduction in the transversal direction. Pacemakers were found to occur in a haphazard manner, never repeating at the same site but ever changing their location and timing [15].

Uterine EMG studies on humans show that power spectrum analysis and propagation speed are able to capture true labor more accurately than the traditionally used clinical techniques [18–19]. All these studies were performed to investigate the EMG signal conduction properties by analyzing the EMG bursts on the whole uterine muscle using multichannel recordings. However, these studies, due to practical reasons, included a limited number of electrodes in different configurations across the abdomen and there is some discrepancy in values of speeds reported between these studies.

Over the past few years we have applied the magnetomyographic (MMG) [20–21] technology to study uterine electrophysiological activity. MMG is a passive technique that has a high-spatial temporal resolution with a large array of sensors. This technology uses biomagnetic field measurement thus making it easier to study the uterine electrophysiology over the entire maternal abdomen compared to placement of EMG electrodes. Uterine MMG [22–23] research has demonstrated its potential in the effort to better understand the mechanism of uterine contraction and we believe that the knowledge obtained from this high-spatial resolution can guide us to translate and interpret meaningful information obtained from the limited sensor EMG recordings. Hence, in this work we recorded MMG signals related to the electrophysiology of the uterus with high-spatial resolution and then split the MMG sensor space over the abdomen into four quadrants or regions. The rationale for this quadrant based approach is based on the fact that this will allow for more accurate comparison with recordings from EMG electrodes in the literature. A high dimension cross-correlation method with a center of gravity (CoG) approach was applied to calculate the delay between pairs of quadrants. Then, the propagation speed was calculated as the ratio of the distance between the CoGs and delay [24]. We also characterized the propagation activity based on the percent of sensors active in a quadrant and the connectivity between the quadrants.

## Methods

### Subjects and Ethics Statement

The study was approved by University of Arkansas for Medical Sciences (UAMS) Institution Review Board and subjects were recruited after they were provided with details of the study in order to obtain an informed signed consent. We enrolled 36 subjects who presented themselves in triage unit of Labor and Delivery and were undergoing monitoring and evaluation of labor.

### Recordings

The data were recorded with a sample rate of 250 Hz using 151 channels from the SARA (SQUID Array for Reproductive Assessment) system installed at UAMS, Little Rock, USA. All the subjects were asked to lean forward and sit comfortably with the sensor array covering their pregnant abdomen. The duration of a recording was typically around 20 minutes. To obtain the MMG signals, we down-sampled the original data to 32 Hz, then we applied a bandpass filter (0.1–1 Hz) to attenuate the interfering maternal and fetal cardiac signals. A notch filter (0.25–0.35 Hz) was applied to suppress the maternal breathing which is a prominent signal around the frequency of 0.33 Hz. Furthermore, we excluded segments with maternal movement using the method described in Govindan et al [25]. The contractile patterns of the MMG signals for each sensor were automatically detected using Wavelet and Hilbert transforms [26].

In order to study the spread of the activity, we divided the sensor space into 4 regions or quadrants and created pairwise combinations (*Q*1 − *Q*2, *Q*1 − *Q*3, *Q*1 − *Q*4, *Q*2 − *Q*3, *Q*2 − *Q*4, *Q*3 − *Q*4). In a next step, we used a 30 s sliding non-overlapping window and identified segments wherein contractile activity was present. We used only those windows for delay analysis when more than 20% of the sensors in that quadrant showed a contractile pattern. These windows were then labelled as active. In each active window, we computed the center of gravities (CoG) for a given sample in each quadrant. In each quadrant, CoG quantifies the spatial location of the MMG activity in a weighted average sense at a given time point. For a given 30-second period, we calculated delay between the CoG from two active windows using time shifted cross-correlation analysis as described in Furdea et al [24]. The delays were tabulated and tested for significance using a bootstrap approach [27]. Finally, the propagation speed was calculated as the ratio of the distance between the average CoGs of the two quadrants to time delay.

**Fig 1:**
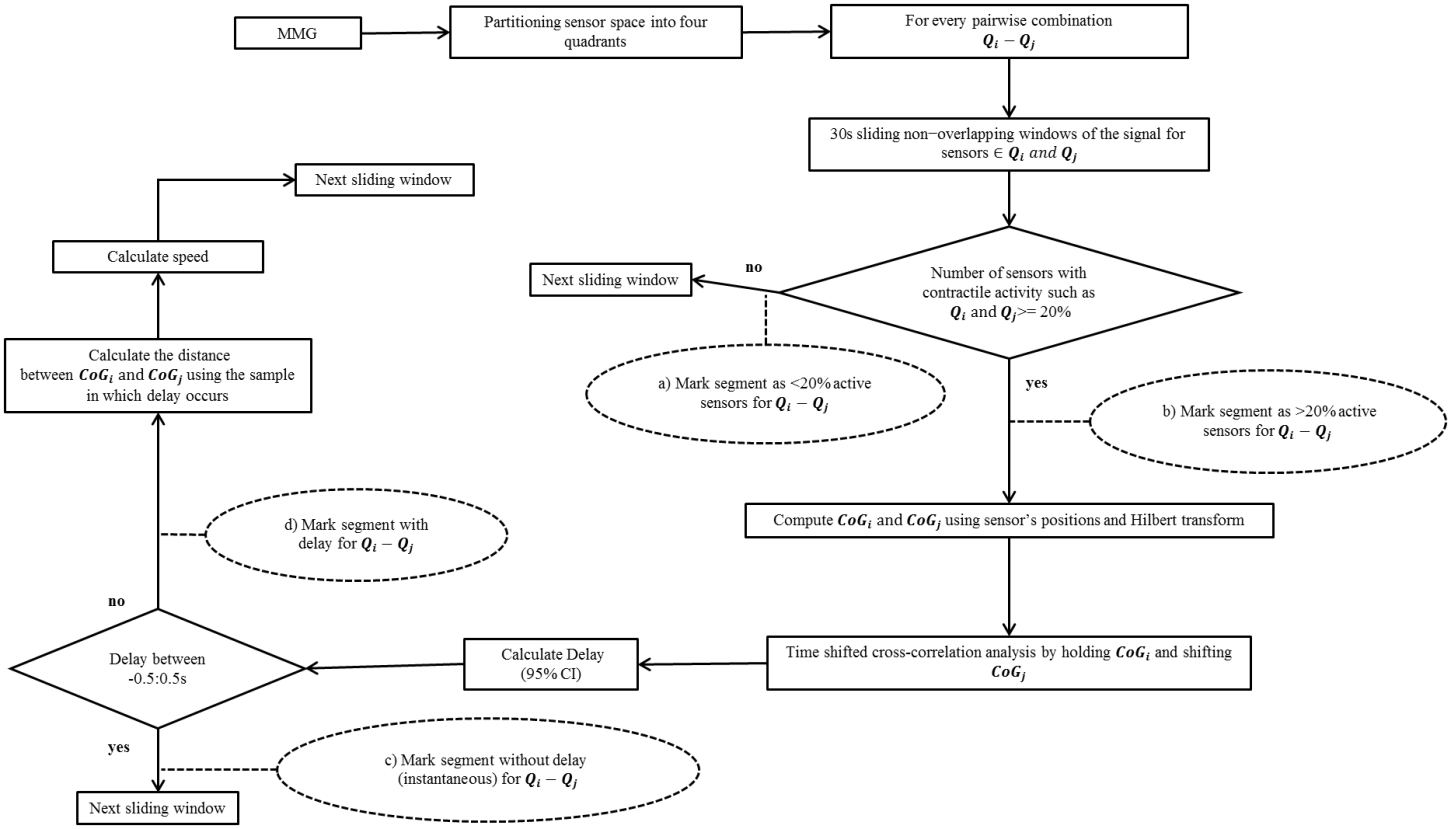
Schematic representation of the propagation speed algorithm. The rectangular boxes describe the flow process and the dotted oval shapes provide the information that was marked or recorded.

Fig 1 shows the flowchart of the algorithm that was applied for quantifying the activity between quadrants and computing the propagation speed. The number of 30 s segments based on the presence of uterine contractile activity to a given threshold in the algorithm for each pairwise combination of quadrants was classified as follows:

- *< 20% active segments*: at least one but less than 20% of the sensors from both quadrants contain contractile activity simultaneously in the pairwise combination: see the dashed oval with item *a)* shown in Fig 1;
- *> 20% active segments*: more than 20% of the sensors from both quadrants contain contractile activity simultaneously in the pairwise combination: see the dashed oval with item *b)* shown in Fig 1;

The propagation speed was computed only for the delays that were outside the range of -0.5 to 0.5 s. The delays in this range of—0.5 to 0.5 s were classified to be segments with “instantaneous” delays in order to avoid obtaining unrealistically high or infinite values for speed. The classification was defined as follows:

- *Segments with delay*: delay was detected after time shifted cross-correlation analysis, see the dashed oval with item *c)* shown in Fig 1;
- *Segments without delay*: instantaneous activity if delay was detected in the interval of -0.5 to 0.5 s, see the dashed oval with item *d)* shown in Fig 1.

For analysis purposes, we used the absolute values in case of negative delay. For comparisons between quadrant pairs, we used Kruskal-Wallis analysis and Tukey’s least significant difference correction with 99% CI.

## Results

Out of the 36 recordings from pregnant subjects, we successfully processed 25 data sets. Eleven datasets were excluded for the following reasons: five had no detectable uterine MMG burst activity during the recording period and six were excluded due to technical reasons (2: metallic interference from the subject; 2: incomplete recordings due to maternal compliance; 2 error in data transfer). The gestation age, cervical dilation prior to the study, time to delivery after recording and total length of data analyzed after post-processing is shown in Table 1. The gestation ages ranged from 37 weeks to a maximum of 40 weeks with 15 subjects delivering ≤ 3 days from the SARA measurement.

**Table 1:** Characteristics of subjects analyzed in the study.

Subject ID	GA (weeks/days)	Duration of recording analyzed (minutes)	Cervical dilation(cm) /effacement %/station	Days to delivery after SARA recording
202	38w0d	17	2-3/50/-3	1
203	37w3d	15	2/25/high	24
204	37w6d	18.5	3-4/50/-2	2
205	40w1d	14	3-4/50/-2	0
207	38w2d	17	1-2/25/-3	4
209	37w3d	18	3/50/-3	10
210	37w3d	18	4/*/*	0
211	37w2d	17	1-2/thick/high	12
212	39w0d	14	2-3/50/-2	1
213	37w4d	14	ft/thick/high	18
214	37w3d	13	ft/thick/high	2
218	38w0d	14	Cesarean/Section	0
221	37w0d	17	1/25/-3	5
222	38w0d	17	3/75/-2	1
224	39w0d	17.5	1-2/75/-3	1
225	37w6d	11.5	closed/50/-3	8
226	38w0d	15.5	2/50/-2	2
227	40w3d	17.5	3/50/-2	1
229	39w1d	15.5	3-4/75/0	1
230	38w5d	17.5	4/50/-2	0
232	37w6d	17.5	4/50/high	1
233	39w3d	16.5	2-3/25/-3	3
234	37w5d	18	3-4/90/-1	0
235	39w0d	10.5	3/50/-2	7
237	40w2d	16.5	ft/thick/high	6

**Fig 2:**
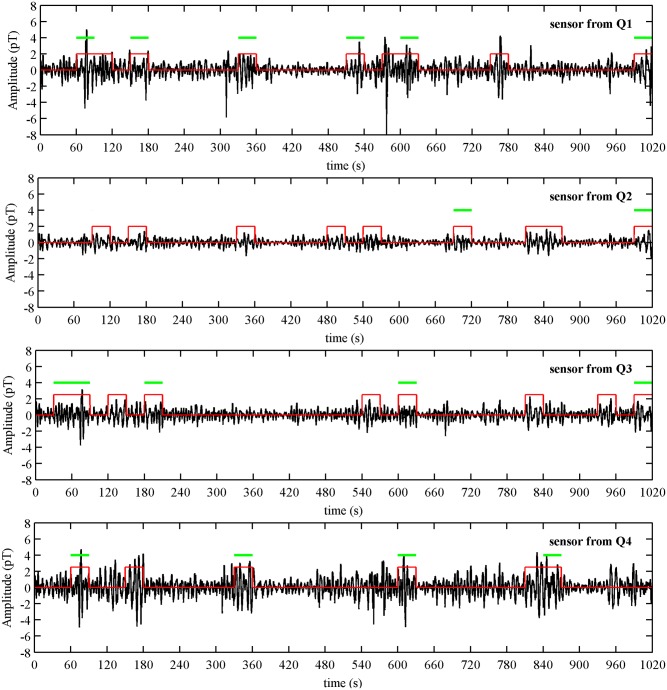
An example of uterine MMG burst activity estimates detected from one sensor in each quadrant for Subject 202. A marker is plotted over burst activity of the MMG signal. The horizontal bars on top indicate the burst activity that fitted the connectivity criteria for the representative sensor shown.

**Fig 3:**
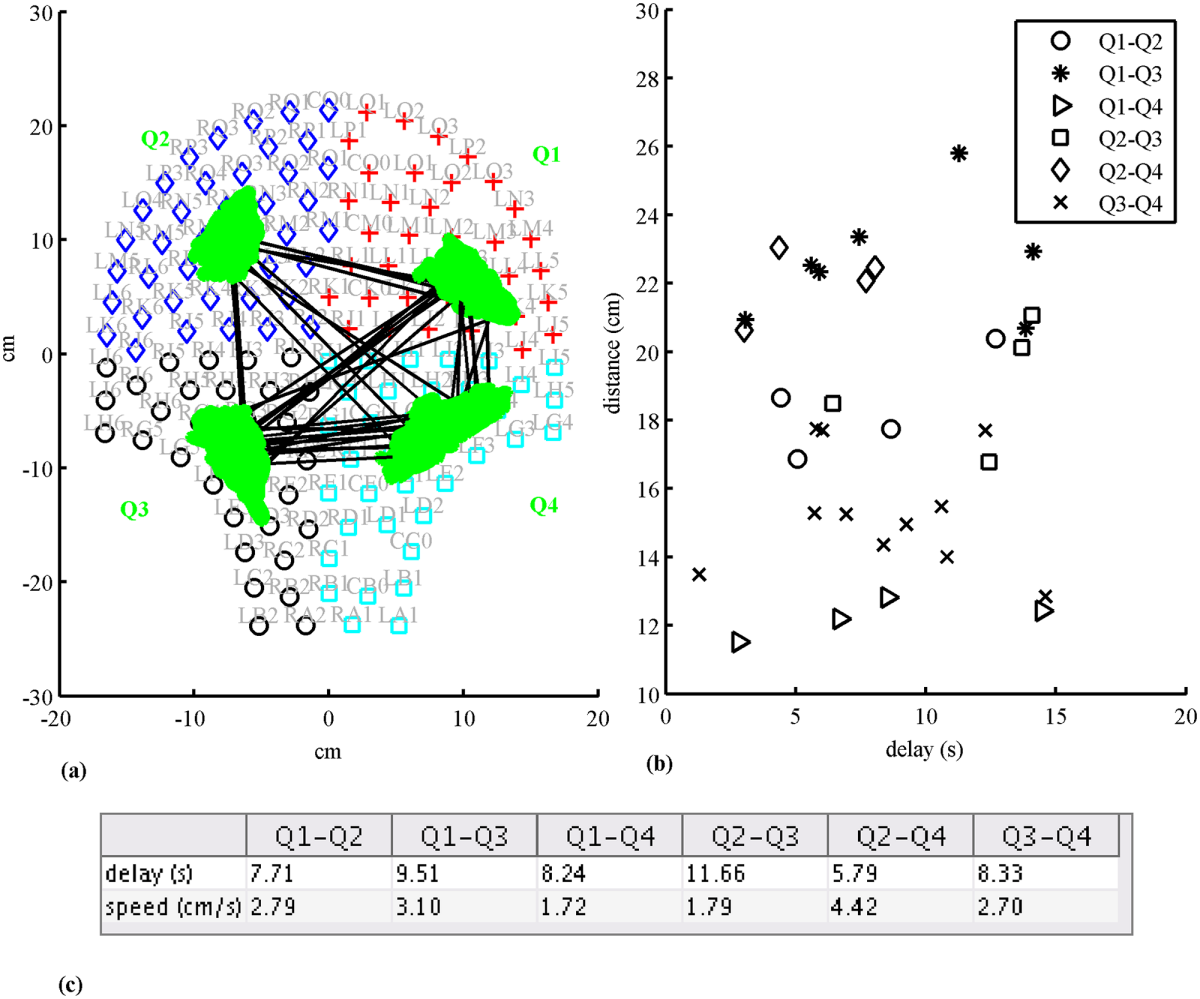
**(a)** Front view of the SARA sensors and partitioning in four quadrants. An example of connectivity (lines) between quadrants and COGs (green dots) for Subject 202. **(b)** Plot showing the distance between COGs between quadrants and detected delay for each connection. **(c)** The table below provides the average values of delay and propagation speeds between quadrants.

Key- Dilation: ft—fingertip to 10; Effacement- thick to100%; Station: -3 to 3; >3—high;

*Data not available;

Fig 2 shows an example of MMG burst activity from a 38 GA weeks subject with cervical dilation of 2 cm recorded one day prior to delivery. The plot is a representation of one channel from each of the four quadrants with the duration of contractile burst activity marked in the figure. In addition, the burst activity that fitted the connectivity criteria for each representative channel in given quadrant is indicated with a horizontal bar. Fig 3a shows the connectivity between quadrants for this recording whenever there was an observable delay in propagation in a given contractile segment across a pair of quadrants. Each line represents a distinct propagation with delay between the centers of activity of each quadrant. In this example majority of the connections appears to link quadrants Q1, Q3 and Q4. The corresponding average values of delays and propagation speeds are shown below the figure. Fig 3b shows the spread of distances between the CoGs for each quadrant pair versus delay for each connection between the quadrants for which a propagation speed was calculated. It can be seen from the figure that the across quadrant pairs had the higher values of distances as compared to the horizontal and vertical pairs. The delay values ranged from 1–16 s and the distances of propagation ranged from 11–26 cm in this example.

**Fig 4:**
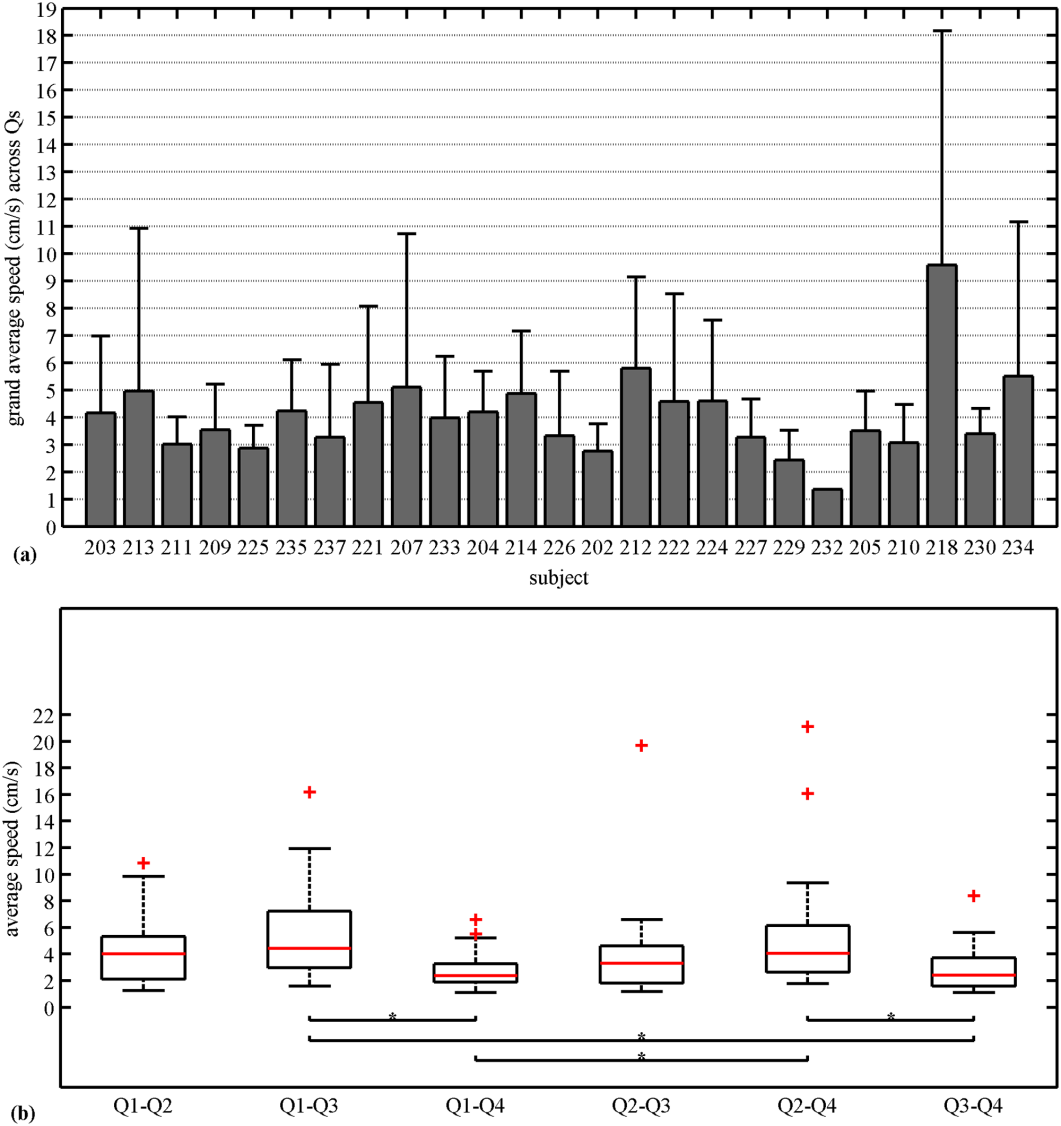
Propagation speed. **(a)** Grand average speed along with standard deviation across quadrants for each subject. **(b)** Average speed for each pairwise combinations of quadrants across subjects. The solid horizontal line inside the box corresponds to median, the edges of the box are the 25th and 75th percentiles, the whiskers extensions are the most extreme data points, and “+” markers correspond to the outliers. Kruskal-Wallis analysis and Tukey’s least significant difference correction with 99% CI was applied.

The grand average and standard deviation of speed for each of the 25 subjects for all the pairwise combinations are shown in Fig 4a. The average values ranged from 1.3–9.5 cm/s. Fig 4b shows the average speed in each quadrant pair across subjects and the median of this quantity as marked in the box plot has the following values in cm/s: 4.03 (Q1-Q2); 4.44 (*Q1-Q3*); 2.36 (*Q1-Q4*);3.30 (*Q2-Q3*); 4.06 (*Q2-Q4*); 2.36 (*Q3-Q4*). After the multiple pairwise test (99% CI), we found no significant differences between horizontal (i.e., *Q*1 −*Q*2 and *Q*3 −*Q*4), vertical (i.e., *Q*1 −*Q*4 and *Q*2 −*Q*3) or crossed pairwise combinations (i.e., *Q*1 − *Q*3 and *Q*2 − *Q*4). However, significant differences can be observed between one of the vertical pair *Q*1 − *Q*4 with the crossed pair combinations, *Q*1 − *Q*3 and *Q*2 − *Q*4. Also, one of the horizontal pairwise *Q*3 − *Q*4 combination is significantly different from both the crossed combinations.

**Fig 5:**
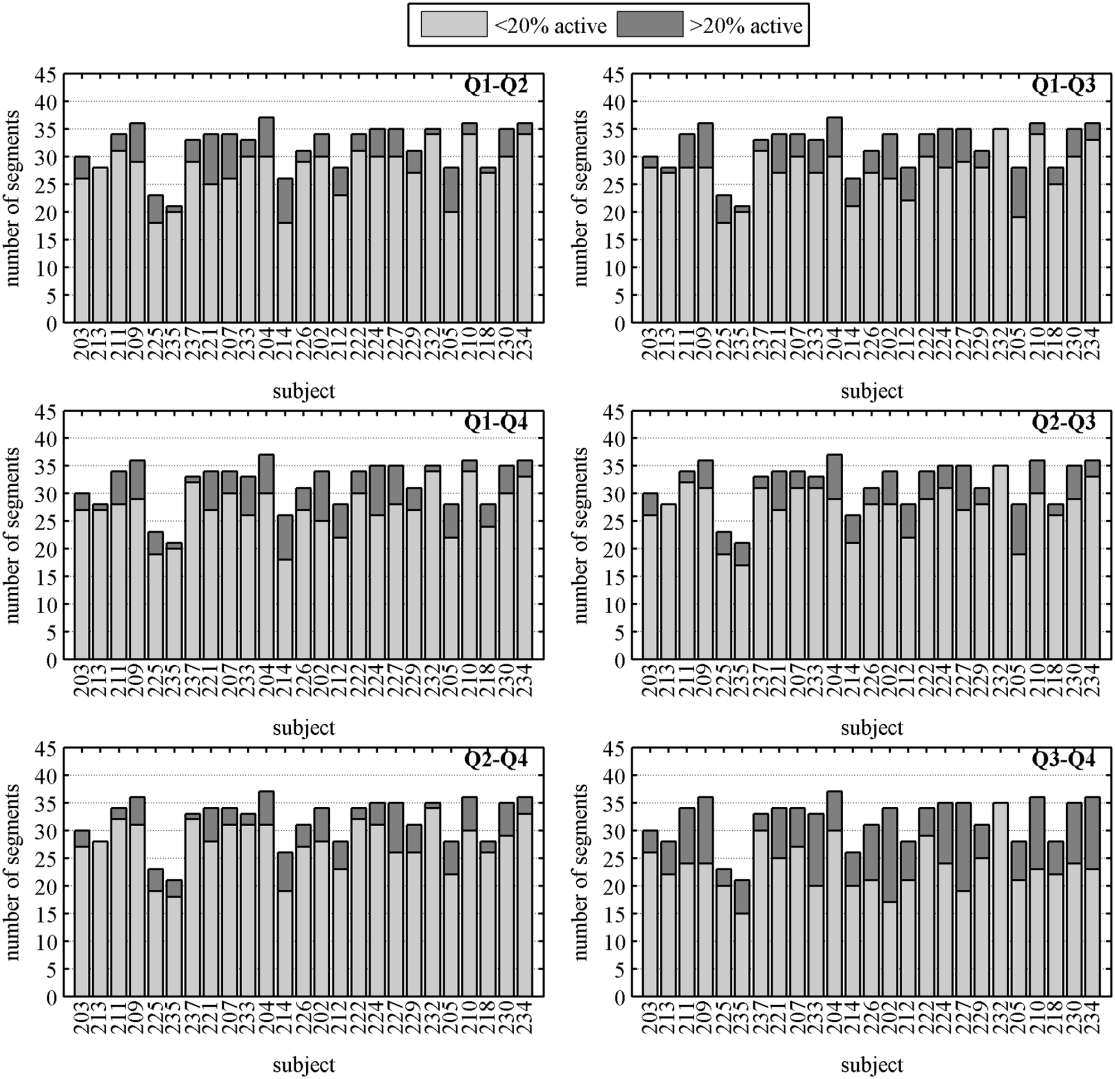
Number of segments that contain less than and more than 20% of active sensors with contractile activity for each pairwise combinations of quadrants per subject.

The quantification of uterine activity across the abdomen in each quadrant is shown in Fig 5. The figure shows stacked bar plots of the number of segments with contractile activity in less than (gray) and more than (dark) 20% of active sensors for each subject in different quadrant pairs. For the case of>20% of active sensors (dark bars) we observed a higher number of segments with contractile activity in *Q*3 − *Q*4.

**Fig 6:**
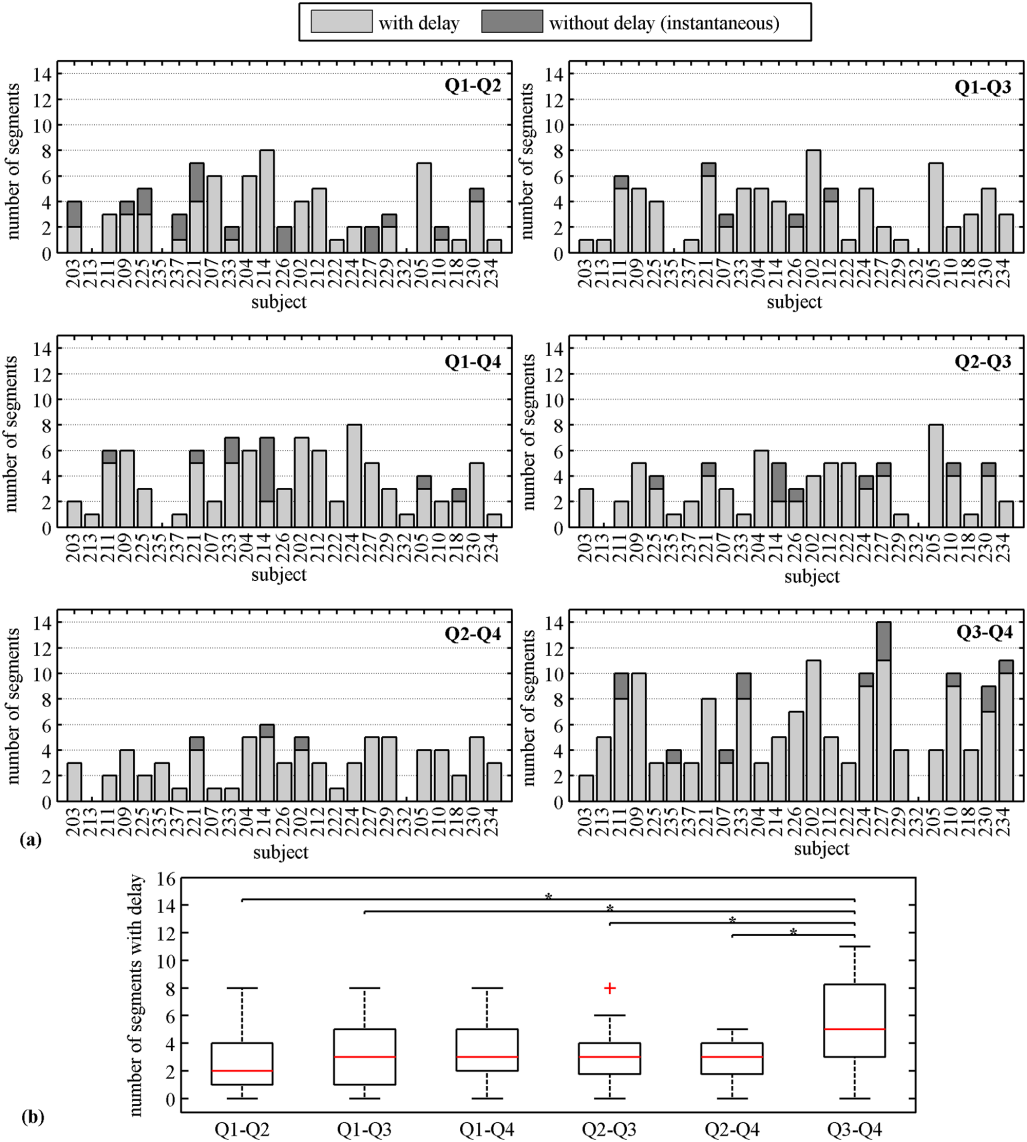
Number of 30 s segments containing contractile activity observed with and without delay in propagation. **(a)** Number of segments with and without delay for each pairwise combinations of quadrants per subject. **(b)** Number of segments with delay for each pairwise combinations of quadrants across subjects. The solid horizontal line inside the box corresponds to median, the edges of the box are the 25th and 75th percentiles, the whiskers extensions are the most extreme data points, and “+” markers correspond to the outliers. Kruskal-Wallis analysis and Tukey’s least significant difference correction with 99% CI was applied.

Fig 6a shows stacked bar plots of the number of segments with and without delay in propagation for each subject for all pairwise combinations. Again in this case, we observed higher number of segments with delay (light bars) in *Q*3 − *Q*4 combination. Fig 6b shows the number of segments with delay across all subjects in each of the quadrants pairs. Based on the multiple pairwise test (99% CI), we found significant difference in the number of segments with delay between *Q*3 − *Q*4 and all other quadrant pairs except with *Q*1 − *Q*4 combination.

## Discussion

Based on our quadrant approach we quantified the uterine electrophysiological activity across the abdomen. Using this approach, we are able to determine the delay and propagation speeds such that it aids in providing a meaningful comparison to existing abdominal EMG studies. Several estimates of the propagation speed have been reported in the human uterus using EMG electrodes applied on the maternal abdomen with various configurations. Recently, Mikkelsen et al [28] reported median speed values of 2.15 (0.66; 13.8) cm/s in the upper part and 1.53 (0.58; 6.7) cm/s in the lower part of the uterus (values in parenthesis represent lower and upper 10th percentiles) using three electrodes along the vertical median axis. The study concluded that the propagation direction occurs both downward and upward, suggesting multidirectional propagation. In a further study by the same group, using 16 electrodes with a 4×4 grid and an inter-electrode distance of 3.5 cm, Lange et al [29] reported speeds of 2.18±0.68 cm/s. In this study too, they did not observe, any single preferred direction of propagation. With our four-quadrant approach the average values of speed in our MMG recordings fall within the same range as reported by them with propagation observed between both vertical and horizontal quadrants confirming their report on multidirectional propagation.

In an earlier study Rabotti et al [19] reported the direction and propagation speed of single action potentials using 8×8 high-density electrode grid located in a small area of the abdominal surface. They obtained values of 3.68 ± 3.24 and 3.76 ± 3.21 cm/s for vertical and horizontal components of the amplitude of the speed, respectively, as well as they found no directional pattern even within the same contraction. Although we used a 30 s windowing approach rather than single action potential to find speed, we also did not observe any significant differences in speeds in propagation in either horizontal or vertical pairs. The difference we observed in the cross-pairs with reference to certain horizontal or vertical pairs is related to the fact that the distances the propagation traversed in the cross-pair centers of activity (i.e. CoG’s) were generally much higher as is evident in the example shown in Fig 3a. As seen in the figure the values related to the distances between the cross quadrants Q1-Q3 and Q2-Q4 are greater than 20 cm as compared to the horizontal or the vertical ones. The fact that the cross-pairs have higher distances appear to be reasonable if we approximate the maternal abdomen (or the uterus) close to a spherical shape then the distances between the CoG’s would reflect values measured along the circumference of the sphere.

There have been only two studies that have attempted to evaluate propagation speed by correlating it with labor characteristics and clinical outcomes. A study by Lau et al [30] reports that the average amplitude of the speed was significantly higher for the labor group (8.65 ± 1.90 cm/s) compared to the non-labor group (5.30 ± 1.47 cm/s). They also showed a high variability of the propagation direction between groups. The other study by Lucovnik et al [18] recorded EMG signals with pairs of electrodes arranged around the navel in four corners with an inter-electrode distance of 2.5 cm. They reported speed values of 31.25 ± 14.91 cm/s and 11.31 ± 2.89 cm/s for labor and non-labor term subjects, respectively and 52.56 ± 33.94 cm/s and 11.11 ± 5.13 cm/s for preterm subjects delivering within and after 7 days from measurement, respectively. There is a significant discrepancy in speed values when comparing the labor vs non-labor groups which were reported in both studies. Further, in general the speeds reported in this last study appear to be higher than all the other EMG studies (described above) and our current MMG study. A plausible explanation of this difference could be attributed to the differences in electrode configuration [31] but all the other human EMG studies obtain values within the expected physiological range similar to the values reported by Lammers et al [15,32] on the intact pregnant guinea pig uterus.

The speed values obtained in our study are based on high-resolution recording reduced to a four-quadrant with weighted COG approach and can be considered to mimic a semi-adaptable EMG configuration based on activation of given area constrained within each quadrant. Several possible explanations have been proposed to explain the high speeds reported by Lucovnik et al [18]. Rabboti et al [31] suggested that this discrepancy is due the combination of the recording methods and the nature of the activity propagation. Also they state that the high speeds are a result of only two channel recordings (four electrodes coupled in bipolar fashion). This explanation may be reasonable based on our results since the four MMG quadrants can be thought of as four electrodes recorded in a unipolar fashion (since magnetic field recordings are independent of references). In addition, in a recent report using a mathematical model based on the concept of “mechanotransduction”, Young et al [33] suggest that the high speed values could be an artifact of recording. They argue that these values probably relate to mechanotransduction occurring nearly at the same time in the case where two regions are independently active and with electrodes that are positioned relatively far apart yielding high speed values. Furthermore, they attribute low speeds values to the action potential propagation within a region rather than across regions. In the case of Lange et al [29] and our measures with the largest distances being across the diagonals (approx. 20 cm), we still observe relatively low speed values in most cases. On the other hand the hypothesis proposed by Young et al [33] could be relevant if we had not excluded delays that were between -0.5 to 0.5 s for calculating the propagation speed. In such instances we may obtain higher speeds in cases even when the distances are relatively large between centers of activity across the quadrant pairs coupled with shorter delays below 0.5 s. Even with the current threshold settings there were two instances where we observed relatively high speed up 40 cm/sec and a closer inspection showed that the delays were close to the 0.5 s boundary with a distance of 20 cm between the centers of activity. In order to further the understanding of the propagation mechanism, we plan to simultaneous MMG and EMG recording with different electrode configurations. We believe that it is reasonable to assume that for a relatively accurate characterization of speeds we would need at least four channels of EMG activity and the optimal electrode separation distance will be guided by the results of the uterine MMG recordings.

The other interesting finding in our study was related to the fact that the most of the segments with delay were observed in the lower horizontal quadrant pair (Q3-Q4). Although we did not observe any difference in speeds between upper and lower horizontal pair, it implies that a higher number segments from the lower horizontal quadrant contributed to calculation of speed values in comparison to other combinations thus indicating higher instances of propagation along the lower abdomen. This aspect can be further investigated in conjunction with uterine modelling approaches that are currently being studied [34–35] since this observation could be attributed to the differences in fiber orientation and the structure of the lower uterine segment as compared to the fundal area.

There are several limitations that should be discussed regarding our study and MMG technology in general. One of the major limitations of the MMG technology is that any substance that has magnetic properties can interfere with the recordings. We had to exclude two recordings because of interference related to metallic implants or materials that were present in the body of the subject that (i.e. metal dental retainers) that could not be removed at the time of study. Another limitation includes the use of a four quadrant approach over the sensor space rather than splitting them in to smaller regions. The four quadrant approach was undertaken after much debate on what would be the optimal division of units that can be considered taking in to account the methodological limitations surrounding the MMG technique. It should to be noted that unlike EMG, MMG recordings provide a global view of the activity under consideration. Uterine EMG records the secondary currents that are incident at the surface of the electrode whereas MMG records the magnetic field related to the primary current. Furthermore, the magnetic field drops off as the inverse cube of distance and also the sensitivity of the “gradiometer” sensor is dependent on its baseline. Thus, unlike an EMG electrode placed at a single location with incident surface current, each MMG sensors will capture the field in the whole general area thus recording contributions from overlapping sources although with varying amplitude based on sensor distance from the source. Based on these methodological issues, smaller units will be detrimental since a group of neighboring sensors will be recording the same signal thus detecting almost no propagation. In order to avoid such an overlap we choose the quadrant approach along with the center of gravity calculation to mimic more of a regional propagation.

As mentioned before, the propagation speed was computed only for the delays that were outside the range of -0.5 to 0.5 s. Although the threshold could have been set at a lower value, we chose a conservative estimate in order to avoid infinite or unrealistically high calculated values of speed. Since this value can have an impact on the prognostic value of propagation speed, so we gave careful consideration to this aspect and did not discard the segments outside the limit. Rather, we bundled them in the “segment without delay” group (Fig 6). Though the cutoff will have some effect, we believe that the high speeds can still be accounted for since we hypothesize that the number segments without delay would increase as one approaches labor and this, in conjunction with speed values, can be a potential parameter for differentiation between true labor and non-labor patients.

Finally, in regard to correlation of delivery outcomes, the data were recorded from patients who presented themselves to the UAMS Labor and Delivery triage unit with a complaint of uterine contractions. At the time of subject recruitment we were blinded to their true labor status as this had not yet been clinically determined. There is therefore a large variation in the number of days between the recording and delivery. This is especially true with the outliers since these subjects were not close to active labor and were discharged after observation. The idea of this study was to characterize uterine MMG activity in patients who present to Labor and Delivery triage for assessment of labor and not necessarily to relate recordings to patient outcomes. In order to correlate the outcomes of delivery to uterine activity, we would need to perform long-duration monitoring which is not feasible with our current system set up. In this study, we performed a one-time recording of uterine MMG signals in order to develop the methodology for observing and validating the EMG speed measures. We are currently analyzing another group of subjects who started the study at 37 weeks and have weekly recordings to track the changes in speed and synchrony. We plan to correlate the outcomes in this group since we will be able to serially quantify the electrophysiological changes as the uterus goes through as it approaches labor.

In summary, our studies show that high-resolution MMG recordings can guide us in improving the understanding of the uterine electrophysiological mechanism and simultaneously provide better insight in to optimizing uterine EMG recordings for improved diagnosis of labor.
